# CCCG-ALL-2015方案与CCLG-ALL-2008方案治疗青少年急性淋巴细胞白血病的远期疗效对比分析

**DOI:** 10.3760/cma.j.cn121090-20250922-00430

**Published:** 2026-01

**Authors:** 硕 林, 惠 吕, 心怡 李, 尧 邹, 芳 刘, 玉梅 陈, 丽 张, 晔 郭, 俊霞 王, 文钰 杨, 晓凡 竺, 晓娟 陈

**Affiliations:** 1 中国医学科学院血液病医院（中国医学科学院血液学研究所）、血液与健康全国重点实验室、国家血液系统疾病临床医学研究中心、细胞生态海河实验室，天津 300020 State Key Laboratory of Experimental Hematology, National Clinical Research Center for Blood Diseases, Haihe Laboratory of Cell Ecosystem, Institute of Hematology & Blood Diseases Hospital, Chinese Academy of Medical Sciences &Peking Union Medical College, Tianjin 300020, China; 2 天津医学健康研究院，天津 301600 Tianjin Institutes of Health Science, Tianjin 301600, China

**Keywords:** 白血病，淋巴细胞，急性, 青少年, 儿童, 微小残留病, Leukemia, lymphoblastic, acute, Adolescent, Children, Minimal residual disease

## Abstract

**目的:**

比较分析CCCG-ALL-2015方案和CCLG-ALL-2008方案治疗10～18岁的大龄儿童及青少年急性淋巴细胞白血病（ALL）的远期疗效与安全性，探讨影响预后的关键因素。

**方法:**

回顾性分析2008年1月至2020年10月于中国医学科学院血液病医院接受CCCG-ALL-2015方案或CCLG-ALL-2008方案治疗的10～18岁ALL患者临床资料。将CCCG-ALL-2015方案组定义为Ⅰ组，CCLG-ALL-2008方案组定义为Ⅱ组。采用Kaplan‑Meier法计算总生存（OS）率及无事件生存（EFS）率，并进行单因素分析，采用Cox比例风险回归模型进行多因素分析。

**结果:**

共纳入355例患者，Ⅰ组210例，Ⅱ组145例；其中男214例，女141例，B-ALL 286例，T-ALL 69例。Ⅰ组诱导治疗完全缓解率为99.5％，高于Ⅱ组的91.7％（*χ*^2^＝14.596，*P*<0.001）。中位随访时间84（2～206）个月，共89例（25.1％）复发，73例（20.6％）死亡；355例患者预期10年EFS率、OS率分别为（65.9±2.6）％、（79.1±2.2）％。其中Ⅰ组预期10年EFS率、OS率分别为（68.3±3.3）％、（80.7±2.7）％，Ⅱ组预期10年EFS率、OS率分别为（62.6±4.0）％、（76.8±3.5）％，差异均无统计学意义（*P*_EFS_＝0.201，*P*_OS_＝0.305）。Ⅰ组感染事件发生率（54.3％对69.7％，*P*＝0.004）、门冬酰胺酶过敏率（0.9％对9.0％，*P*<0.001）均显著低于Ⅱ组。多因素分析结果显示，初诊WBC≥50×10^9^/L（*HR*＝1.583，95％*CI*：1.060～2.365，*P*＝0.025）和诱导治疗末微小残留病（MRD）≥0.01％（*HR*＝1.300，95％*CI*：1.174～1.439，*P*<0.001）是影响患者EFS的独立危险因素；MLLr融合基因阳性（*HR*＝3.144，95％*CI*：1.093～9.046，*P*＝0.034）和诱导治疗末MRD≥0.01％（*HR*＝1.310，95％*CI*：1.152～1.488，*P*<0.001）是影响患者OS的独立危险因素。

**结论:**

CCCG-ALL-2015方案通过MRD动态风险分层及药物优化，可显著提升10～18岁ALL患者的诱导缓解质量，降低不良事件发生率。诱导治疗末MRD是评估青少年ALL预后的关键指标，对指导个体化治疗具有重要意义。

## Abstract (en)

**Objective:**

To compare the long-term efficacy and safety of the CCCG-ALL-2015 and CCLG-ALL-2008 protocols in treating adolescents and older children（aged ≥10 years）with acute lymphoblastic leukemia（ALL）and explore the key factors affecting prognosis.

**Methods:**

A retrospective analysis was conducted on the clinical data of patients aged 10–18 years who were admitted to the Institute of Hematology & Blood Diseases Hospital, Chinese Academy of Medical Sciences between January 2008 and October 2020. The patients were treated with either the CCCG-ALL-2015（Group Ⅰ）protocol or the CCLG-ALL-2008（Group Ⅱ）protocol. The Kaplan-Meier analysis was used to evaluate the overall survival（OS）and event-free survival（EFS）rates,and for univariate analysis. Multivariate analysis was performed using the Cox proportional-hazards regression model.

**Results:**

Altogether, 355 patients were enrolled, including 210 in Group Ⅰ and 145 in Group Ⅱ. There were 214 men and 141 women; 286 and 69 had B-ALL and T-ALL, respectively. The complete remission rate after induction therapy in Group Ⅰ was 99.5％, which was higher than the in Group Ⅱ at 91.7％（*P*<0.001, *χ*^2^＝14.596）. The median follow-up time was 84（range: 2–206）months. A total of 89 patients（25.1％）relapsed, and 73（20.6％）died. The predicted 10-year EFS and OS rates for all 355 patients were（65.9 ± 2.6）％ and（79.1 ± 2.2）％, respectively. The predicted 10-year EFS and OS rates for Group Ⅰ were（68.3 ± 3.3）％ and（80.7 ± 2.7）％, respectively, whereas those for Group Ⅱ were（62.6 ± 4.0）％ and（76.8 ± 3.5）％, respectively, showing no significant difference（*P*_EFS_＝0.201, *P*_OS_＝0.305）. The rates of infectious events（54.3％ *vs* 69.7％, *P*＝0.004）and asparaginase allergy（0.9％ *vs* 9.0％, *P*<0.001）were significantly lower in Group Ⅰ than in Group Ⅱ. Multivariate analysis identified an initial WBC ≥50×10^9^/L（*HR*＝1.583，95％ *CI*: 1.060–2.365，*P*＝0.025）and minimal residual disease（MRD）value ≥0.01％ at the end of induction therapy（*HR*＝1.300，95％ *CI*: 1.174–1.439，*P*<0.001）as the independent risk factors for EFS. MLL rearrangement（*HR*＝3.144, 95％*CI*: 1.093–9.046，*P*＝0.034）and MRD value ≥0.01％ at the end of the induction therapy（*HR*＝1.310，95％*CI*: 1.152–1.488，*P*<0.001）were the independent risk factors for OS.

**Conclusion:**

By incorporating MRD-guided dynamic risk stratification and optimized drug regimens, the CCCG-ALL-2015 protocol significantly improved the quality of induction remission, reduced adverse events, and improved the long-term survival in adolescents（aged 10–18 years）with ALL. The MRD status at the end of induction therapy is a critical prognostic indicator and provides important guidance for individualized therapy.

急性淋巴细胞白血病（ALL）是起源于淋巴祖细胞的血液系统恶性克隆性疾病[1]，是儿童最常见的恶性肿瘤。随着诊疗方案的不断优化，ALL患儿5年总生存（OS）率可接近90％[2]，而成人ALL患者长期生存率仅为40％～50％，年龄仍是影响ALL预后的关键因素。10岁以上儿童及青少年ALL患者具有独特的生物学特征[3]，其临床特征和治疗反应介于低龄儿童与成人之间[4]。CCCG-ALL-2015方案和CCLG-ALL-2008方案均为我国多中心协作开展的儿科ALL规范化治疗研究方案，显著改善了国内儿童ALL的总体疗效[5]–[6]。本研究通过回顾性分析接受上述方案治疗的10～18岁ALL患者的临床资料，比较其治疗反应和生存差异，并探讨影响预后的危险因素，旨在为大龄儿童及青少年ALL的治疗策略优化提供参考。

## 对象与方法

一、研究对象

本文为回顾性队列研究，收集2008年1月至2020年10月中国医学科学院血液病医院收治的355例10～18岁ALL患儿的临床资料。纳入标准：①年龄10～18岁；②经骨髓细胞形态学、免疫分型、细胞遗传学及分子生物学诊断为ALL；③接受CCCG-ALL-2015方案或CCLG-ALL-2008方案规范治疗，并至少完成诱导缓解治疗。排除标准：①合并先天性免疫缺陷病或其他遗传性疾病；②继发性白血病或其他恶性肿瘤。本研究已通过中国医学科学院血液病医院伦理委员会批准，并豁免知情同意（IIT2021009-EC-1）。

二、方法

1. 治疗方案：2015年5月至2020年10月收治患儿接受CCCG-ALL-2015方案治疗，定义为Ⅰ组；2008年1月至2015年4月收治患儿接受CCLG-ALL-2008方案治疗，定义为Ⅱ组。CCCG-ALL-2015方案[5]及CCLG-ALL-2008方案[6]治疗均包括窗口期、诱导缓解期、巩固治疗期及维持治疗期，两方案主要药物差异在于CCCG-ALL-2015方案增加了门冬酰胺酶剂量并对其剂型进行调整，减少了柔红霉素给药次数，同时BCR::ABL阳性患者确诊起规律应用酪氨酸激酶抑制剂（TKI）治疗，具体用药详见相关文献[5]–[6]。

2. 危险度分层：根据初诊临床特征将患者初步分为低危组、中危组和高危组，结合治疗反应进一步调整，最终确定危险分层。Ⅰ组患者依据诱导治疗后第19天（D19）和第46天（D46）的微小残留病（MRD）水平动态调整危险度分层。本研究患者危险度分层标准见表1。

**表1:** 本研究急性淋巴细胞白血病患者危险度分层

组别	低危组	中危组	高危组
Ⅰ组	1. 必要条件（B-ALL满足以下条件之一）： （1）染色体≥50或DNA指数≥1.16； （2）ETV6::RUNX1 融合基因型 2. 必须排除以下情况： （1）CNS3和（或）睾丸白血病； （2）t（1;19）、t（9;22）、MLLr、染色体<44、iAMP21； （3）第19天MRD≥1％	1. 所有无法归入低危组及高危组B-ALL； 2. 所有无法归入高危组的T-ALL	第46天MRD≥1％
Ⅱ组	无	同时满足以下条件的患儿： 1. 无t（9;22）或BCR::ABL融合基因； 2. 无t（4;11）等MLL融合基因重排； 3. 第8天外周血白血病细胞<1×10^9^/L； 4. 诱导治疗第15天骨髓呈M1/M2； 5. 诱导治疗第33天骨髓形态学缓解； 6. 如行MRD检测，第33天MRD<0.01％	所有非中危组患儿

**注** Ⅰ组：接受CCCG-ALL-2015方案治疗；Ⅱ组：接受CCLG-ALL-2008方案治疗；MRD：微小残留病；CNS3：中枢神经系统状态3

3. 疗效标准及相关定义：复发指患者经治疗获得完全缓解（CR）后外周血再次出现白血病细胞，或骨髓中原始细胞比例≥25％，或出现髓外浸润［中枢神经系统（CNS）、睾丸等］。MRD阴性定义为骨髓MRD<10^−4^；MRD-CR定义为在骨髓达到形态学CR同时MRD<10^−4^。

4. 随访：通过门诊复查、电话及查阅病历方式进行随访，随访截止日期为2025年7月31日。OS期定义为自诊断起至任何原因死亡或末次生存随访时间。无事件生存（EFS）期为自诊断至发生首次不良事件的时间，包括诱导治疗失败、复发、死亡、继发肿瘤、放弃治疗。

5. 统计学处理：采用SPSS 25.0软件进行数据分析。符合正态分布的计量资料以*x*±*s*表示，非正态分布的计量资料以*M*（*Q*_1_，*Q*_3_）表示。计数资料用例数和百分比（％）表示。组间比较采用*χ*^2^检验。生存分析采用Kaplan-Meier法计算EFS和OS，组间差异比较使用Log-rank检验。将单因素分析中*P*<0.05的变量纳入Cox比例风险回归模型进行多因素分析，以双侧*P*<0.05为差异有统计学意义。

## 结果

一、临床特征

本研究共纳入355例新诊断ALL患者，Ⅰ组210例，Ⅱ组145例，其中男214例，女141例。中位年龄12（10～17）岁，B-ALL共286例（80.6％），T-ALL 69例（19.4％）。初诊外周血中位WBC为14.01（0.66～781.60）×10^9^/L，高白细胞血症（WBC≥50×10^9^/L）者107例（30.1％）。初诊脑脊液非CNS1状态患者41例。所有患者均接受融合基因检查，其中ETV6::RUNX1阳性25例、BCR::ABL阳性39例、E2A::PBX1阳性23例、MLL重排（MLLr）阳性患者9例；染色体核型为高二倍体者13例。两组患者在基线特征方面差异均无统计学意义（均*P*>0.05），详见表2。

**表2:** 接受CCCG-ALL-2015方案（Ⅰ组）和接受CCLG-ALL-2008方案（Ⅱ组）患者临床特征比较（例）

临床特征	例数	Ⅰ组（210例）	Ⅱ组（145例）	*χ*^2^值	*P*值
性别					
男	214	130	84	0.566	0.452
女	141	80	61		
初诊WBC					
<50×10^9^/L	248	145	103	0.161	0.688
≥50×10^9^/L	107	65	42		
免疫分型					
T细胞型	69	41	28	0.002	0.960
B细胞型	286	169	117		
初诊CNS状态					
CNS1	314	184	130	0.348	0.555
非CNS1	41	26	15		
融合基因					
ETV6::RUNX1	25	15	10	1.912	0.752
BCR::ABL	39	26	13		
E2A::PBX1	23	15	8		
MLLr	9	6	3		
染色体					
高二倍体	13	7	6	0.157	0.692
非高二倍体	342	203	139		

**注** CNS：中枢神经系统

二、临床疗效及安全性分析

1. 早期治疗反应及长期生存分析：全组355例患者中，5例患者诱导治疗结束未进行骨髓评估，该部分患者均死亡，中位OS期为2（2～13）个月；其余350例患者均完成诱导治疗末骨髓评估，3.7％（13/350）患者未达骨髓细胞形态学CR，其3年OS率为（38.5±13.5）％。Ⅰ组骨髓CR率为99.5％（205/206），显著高于Ⅱ组的91.7％（132/144），差异有统计学意义（*χ*^2^＝14.596，*P*<0.001）。Ⅰ组MRD-CR率为77.7％（160/206），亦高于Ⅱ组的63.2％（86/136），差异有统计学意义（*χ*^2^＝8.453，*P*＝0.004）。

全组355例患者中位随访84（2～206）个月，355例患者5年EFS率和OS率分别为（68.0±2.5）％、（79.8±2.1）％，预期10年EFS率和OS率分别为（65.9±2.6）％和（79.1±2.2）％。其中Ⅰ组预期10年EFS率和OS率分别为（68.3±3.3）％和（80.7±2.7）％；Ⅱ组预期10年EFS率、OS率为（62.6±4.0）％、（76.8±3.5）％。Ⅰ组远期生存率高于Ⅱ组，但差异无统计学意义（*P*_EFS_＝0.201, *P*_OS_＝0.305）；生存分析显示，Ⅰ组患儿于63个月后进入生存平台期，Ⅱ组患儿于72个月进入生存平台期，患者生存曲线见图1～2。

**图1:**
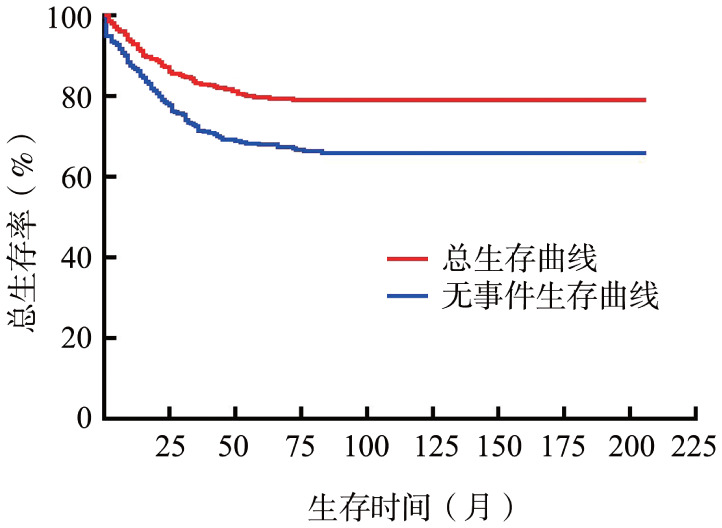
355例大龄儿童及青少年急性淋巴细胞白血病患者的生存曲线

**图2:**
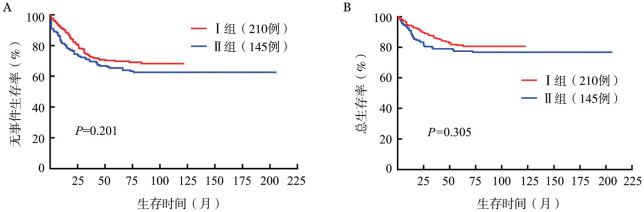
两组大龄儿童及青少年急性淋巴细胞白血病患者的无事件生存曲线（A）、总生存曲线（B）比较 **注** Ⅰ组：接受CCCG-ALL-2015方案治疗；Ⅱ组：接受CCLG-ALL-2008方案治疗

2. 复发及死亡情况：共89例（25.1％）患者复发，其中单纯骨髓复发76例，单纯CNS复发9例，单纯睾丸复发1例，骨髓联合CNS复发2例，骨髓联合睾丸复发1例。Ⅰ组复发率为25.2％（53/210），Ⅱ组复发率为24.8％（36/145），差异无统计学意义（*P*＝0.811），复发患儿情况详见表3。患者中位复发时间为21（3～83）个月，64例复发后接受再诱导治疗，其中30例接受造血干细胞移植（HSCT）。复发患者5年OS率为（40.7±5.3）％，其中接受HSCT患者5年OS率为（70.0±8.4）％，显著高于未行HSCT组患者的（25.4±5.8）％（*χ*^2^＝16.904，*P*<0.001）。

**表3:** 89例大龄儿童及青少年急性淋巴细胞白血病（ALL）复发患儿情况（例）

复发时间	例数	危险度			免疫分型		融合基因				复发部位		
		低危	中危	高危	T-ALL	B-ALL	ETV6::RUNX1	BCR::ABL	E2A::PBX1	MLLr	骨髓	髓外	联合
<18个月	36	0	21	15	7	29	0	3	2	4	35	0	1
18～36个月	36	2	30	4	4	32	1	3	2	0	27	7	2
>36个月	17	0	13	4	2	15	1	3	1	0	14	3	0

合计	89	2	64	23	13	76	2	9	5	4	76	10	3

本研究患者死亡率为20.6％（73/355），其中Ⅰ组死亡率19.0％（40/210），31例因疾病复发死亡，5例为感染相关死亡，1例未缓解后死亡，3例放弃治疗。Ⅱ组死亡率22.8％（33/145），23例因疾病复发死亡，4例为感染相关死亡，4例未缓解后死亡，2例放弃治疗。

3. 化疗不良反应：本研究统计患者化疗期间的主要不良反应，包括感染事件（肺部感染、消化道感染、血流感染及感染性休克）、肿瘤溶解综合征、血栓/出血事件（颅内血栓、外周血管血栓，消化道出血、颅内出血、肺出血）、门冬酰胺酶过敏、继发性高血糖/糖尿病和门冬酰胺酶相关性胰腺炎（Asparaginase-associated pancreatitis，AAP）。感染事件是最常见的不良反应，Ⅰ组感染事件发生率和门冬酰胺酶过敏率显著低于Ⅱ组，差异均具有统计学意义（均*P*<0.05）。其余不良反应发生率两组间差异均无统计学意义，两组各类不良反应发生情况详见表4。

**表4:** 接受CCCG-ALL-2015方案（Ⅰ组）和接受CCLG-ALL-2008方案（Ⅱ组）患者化疗不良反应发生情况［例（％）］

不良反应	总例数	Ⅰ组	Ⅱ组	*χ*^2^值	*P*值
全疗程感染事件	215（60.6）	114（54.3）	101（69.7）	8.483	0.004
肿瘤溶解综合征	10（2.8）	5（2.4）	5（3.4）	0.357	0.550
血栓/出血事件	30（8.4）	19（9.0）	11（7.6）	0.237	0.627
门冬酰胺酶过敏	15（4.2）	2（0.9）	13（9.0）	13.610	<0.001
门冬酰胺酶相关性胰腺炎	19（5.4）	14（6.7）	5（3.4）	1.754	0.185
继发性高血糖/糖尿病	13（3.7）	10（4.8）	3（2.1）	1.763	0.184

三、预后因素分析

单因素分析结果显示，初诊WBC、肝脾肿大、ETV6::RUNX1融合基因、MLLr、诱导治疗中期MRD水平、诱导治疗末MRD水平及最终危险度分层与患儿EFS及OS显著相关（均*P*<0.05），此外窗口期激素治疗敏感性与EFS显著相关（*P*<0.05）（表5），将上述因素纳入Cox比例风险回归模型行多因素分析。结果显示，初诊WBC≥50×10^9^/L（*HR*＝1.583，95％*CI*：1.060～2.365，*P*＝0.025）和诱导治疗末MRD≥0.01％（*HR*＝1.300，95％*CI*：1.174～1.439，*P*<0.001）是影响患者EFS的独立危险因素；MLLr阳性（*HR*＝3.144，95％*CI*：1.093～9.046，*P*＝0.034）和诱导治疗末MRD≥0.01％（*HR*＝1.310，95％*CI*：1.152～1.488，*P*<0.001）是影响患者OS的独立危险因素。上述结果提示，MRD水平仍是评估大龄儿童及青少年ALL预后的关键指标。

**表5:** 355例大龄儿童及青少年急性淋巴细胞白血病（ALL）患者的预后分析

因素	例数	5年EFS率（％）	*χ*^2^值	*P*值	5年OS率（％）	*χ*^2^值	*P*值
性别			0.068	0.794		0.479	0.489
男	214	67.3±3.2			78.8±2.8		
女	141	69.2±3.9			81.3±3.3		
初诊WBC			15.324	<0.001		10.079	0.001
<50×10^9^/L	248	74.1±2.8			83.7±2.4		
≥50×10^9^/L	107	53.9±4.8			70.4±4.5		
免疫分型			0.012	0.913		0.952	0.329
B细胞型	286	68.0±2.8			80.8±2.3		
T细胞型	69	68.1±5.6			75.4±5.2		
初诊CNS状态			0.562	0.453		0.049	0.826
CNS1	314	68.7±2.6			80.0±2.3		
非CNS1	41	62.8±7.6			77.7±6.6		
染色体核型			0.081	0.776		0.044	0.834
高二倍体	13	69.2±12.8			76.9±11.7		
非高二倍体	342	68.0±2.5			79.9±2.2		
初诊时肝大			5.360	0.021		5.575	0.018
是	143	59.9±4.1			74.0±3.7		
否	212	73.5±3.1			83.7±2.6		
初诊时脾大			5.288	0.021		5.299	0.021
是	185	62.0±3.6			75.5±3.2		
否	170	74.6±3.3			84.4±2.8		
伴ETV6::RUNX1			5.059	0.024		6.306	0.012
是	25	88.0±6.5			100.0±0.0		
否	330	66.5±2.6			78.2±2.3		
伴BCR::ABL1			0.476	0.490		0.152	0.697
是	39	61.5±7.8			81.6±6.3		
否	316	68.9±2.6			79.5±2.3		
伴E2A::PBX1			0.641	0.423		0.156	0.693
是	23	73.9±9.2			86.7±7.1		
否	332	67.6±2.6			79.3±2.3		
伴MLLr			4.696	0.030		12.449	<0.001
是	9	44.4±16.6			44.4±16.6		
否	346	68.6±2.5			80.7±2.1		
窗口期激素治疗			11.074	0.001		3.812	0.051
敏感	218	73.8±3.0			83.4±2.5		
不敏感	137	58.9±4.2			73.9±3.8		
诱导治疗中期MRD			5.545	0.019		4.576	0.032
<0.01％	98	75.5±4.3			87.7±3.3		
≥0.01％	244	64.6±3.1			76.7±2.7		
NA	13	–			–		
诱导治疗末MRD			47.085	<0.001		32.166	<0.001
<0.01％	246	77.6±2.7			88.1±2.1		
≥0.01％	96	47.3±5.1			62.4±5.0		
NA	13	–			–		
最终危险分层			26.234	<0.001		20.511	<0.001
低危	18	83.3±8.8			94.4±5.4		
中危	258	73.1±2.8			84.4±2.3		
高危	75	50.4±5.8			64.3±5.6		
NA	4	–			–		

**注** EFS：无事件生存；OS：总生存；CNS：中枢神经系统；MRD：微小残留病；NA：缺失数据，未纳入统计；–：无资料

## 讨论

ALL是儿童最常见的造血系统恶性肿瘤[7]，随着诊疗体系及方案优化，儿童ALL患者5年OS率可达90％[2]，但10～18岁的大龄儿童及青少年ALL患者在临床表现、遗传学特征及治疗反应方面均与低龄患儿存在显著差异[8]，国内青少年ALL患者5年OS率为68％～79％[4],[9]–[10]，提示该群体仍需特别关注。CCCG-ALL-2015方案在CCLG-ALL-2008方案基础上，通过MRD指导的动态风险分层和药物优化进一步提升疗效[11]。本研究回顾性分析355例10～18岁ALL患者的临床资料，比较两代治疗方案的疗效及安全性，并探讨影响预后的相关因素。

研究结果显示，Ⅰ组患者诱导治疗末CR率及MRD阴性缓解率显著高于Ⅱ组，实现了更高效的早期肿瘤清除和深度骨髓缓解，并更早进入生存平台期，预期10年OS率达（80.7±2.7）％，兼顾了快速起效与长远获益。CCCG-ALL-2015方案针对不同风险群体优化了药物强度与剂型，总体增加门冬酰胺酶的使用剂量，中高危组用培门冬酶（PEG-Asp）取代常规门冬酰胺酶，低危组诱导期门冬酰胺酶增加到10次。PEG-Asp作为门冬酰胺酶的聚乙二醇化长效剂型，免疫原性低、半衰期长，可降低门冬酰胺酶过敏事件发生率，适合作为青少年ALL患者一线门冬酰胺酶用药[12]，但需警惕AAP风险并加强对血淀粉酶、脂肪酶监测。蒽环类药物是ALL化疗中骨髓毒性最大的药物之一，其心脏毒性与累积量相关[13]。CCCG-ALL-2015方案对柔红霉素进行合理的剂量下调：将诱导期柔红霉素剂次由CCLG-ALL-2008方案的4次减至2次（单次25 mg/m²），同时减少全疗程给药次数，低危组总剂量仅为75 mg/m^2^，中高危组总剂量由CCLG-ALL-2008方案中的250～260 mg/m^2^降至175 mg/m^2^，在不影响诱导疗效和长期生存的前提下，减少了骨髓抑制和心脏毒性[14]。化疗期间，感染是患儿最常见的不良反应，CCCG-ALL-2015方案的感染事件发生率较CCLG-ALL-2008方案显著下降，提示在方案设计和支持治疗优化的共同作用下，感染控制得到改善，但仍需继续强化早期识别与干预，进一步改善患儿预后。

青少年ALL患者常伴高比例的预后不良因素。本研究中，BCR::ABL阳性患者比例11.0％，在TKI时代前，该亚组患者预后极差[15]。两代方案核心差异之一在于CCCG-ALL-2015方案对该类患者早期、规范应用小分子靶向药物TKI治疗，并将风险分层调整至中危，降低了HSCT需求并改善了未移植患者远期生存[16]。ALL患者中T-ALL患者占19.4％，5年OS率为75.4％，较既往有所改善，但诱导治疗末MRD阳性者预后较差，建议CR1期积极考虑HSCT[17]。CCCG-ALL-2015方案将治疗反应良好的ETV6::RUNX1阳性青少年ALL患者纳入低危组，疗效安全可靠[14]。本研究表明，初诊高白细胞血症、MLLr阳性以及诱导治疗末MRD阳性是影响预后的独立危险因素。本研究中MLLr阳性患者多伴高白细胞血症，易发生极早期骨髓复发，复发患儿全部死亡，5年OS率仅为44.4％，提示传统化疗难以满足此类高危患儿治疗需求，亟需新型治疗策略。诱导治疗末MRD阳性是预测预后的最强指标[18]，与较差的EFS和OS显著相关，可早期识别复发风险并指导个体化精准治疗。

复发仍是限制青少年ALL长期生存的关键因素，本研究中复发率达25.1％，明显高于低龄患儿[6],[11]。患者复发后5年OS率仅约40％，是主要死亡原因。HSCT是高危及复发患者的重要治疗手段，但疗效受移植前疾病负荷及移植相关并发症等影响。当前，新型免疫与靶向治疗显示良好前景。在MRD阳性或高危患者CR1期，CAR-T细胞疗法可显著改善疗效[19]；奥加伊妥珠单抗在复发或难治性ALL中再诱导治疗有效[20]，达雷妥尤单抗在T-ALL中展示潜在疗效[21]，Menin抑制剂等[22]为高危亚型提供新治疗方案。贝林妥欧单抗在儿童和青少年B-ALL中应用广泛，可用于无法耐受常规化疗的初治患儿[23]，亦可联合第三代TKI药物[24]用于BCR::ABL阳性患者，进一步降低HSCT需求[25]，并可桥接HSCT治疗改善复发患儿的移植后生存[26]。

综上，CCCG-ALL-2015方案在延续核心化疗框架的基础上，通过MRD导向的动态危险度分层、化疗药物剂量和剂型优化以及分子靶向治疗的整合，提高青少年ALL患者的早期缓解质量并改善了远期生存，为低毒高效的治疗提供依据。未来在治疗过程中需加强对化疗不良反应的监测与早期干预，兼顾青春期患者的身心发展。受限于多药联合的传统化疗方案，高危亚型侵袭性强、复发率高且复发后生存率低仍是治疗的瓶颈问题。当前ALL治疗正迈入精准时代，新型疗法有望突破化疗耐药壁垒，降低化疗毒性并进一步提高疗效。本研究作为单中心回顾性研究，样本量相对有限，仍存在一定的局限性，亟需多中心、前瞻性的研究进一步验证并优化大龄儿童及青少年ALL的治疗策略。
